# Drug-induced PD-L1 expression and cell stress response in breast cancer cells can be balanced by drug combination

**DOI:** 10.1038/s41598-019-51537-7

**Published:** 2019-10-22

**Authors:** Yosi Gilad, Yossi Eliaz, Yang Yu, Sang Jun Han, Bert W. O’Malley, David M. Lonard

**Affiliations:** 1 Department of Molecular and Cellular Biology, Baylor College of Medicine, Houston, Tx USA; 2 Department of Molecular and Human Genetics, Baylor College of Medicine, Houston, Tx USA

**Keywords:** Chemotherapy, Breast cancer, Cancer therapeutic resistance

## Abstract

The impact of chemotherapy on tumor-immune system interaction can be either beneficial or harmful, which is represented by the immunogenic cell death (ICD) paradigm or overexpression of the immunosuppressive protein – programmed death ligand 1 (PD-L1). In this study we explore the impact of steroid receptor coactivator inhibitor, other targeted anti-cancer compounds and traditional chemotherapeutic agents on the expression of PD-L1 in four breast cancer (BC) cell lines. Our results show that these agents induce PD-L1 expression, yet the magnitude of this induction varies substantially across the different compounds. In addition, we utilized the E0771 ER + BC cells as a model to examine in greater detail the relationship between pharmacological pressure, cell stress and the induction of PD-L1. Our results imply that drug induced PD-L1 expression occurs in the broader context of cell-stress, without conferring acquired drug-resistance. Furthermore, a balance between BC cytotoxicity, induction of cell-stress and the overexpression of PD-L1 can be achieved through the selection of appropriate combinations of anti-cancer compounds. Therefore, we propose that drug combination can be employed not only for increasing the direct kill of cancer cells, but also as a strategy to minimize the activation of immunosuppressive and cancer cell pro-survival program responses during drug treatment.

## Introduction

Chemotherapy is one of the three standard treatment modalities for cancer treatment, along with surgery and radiation. In the last two decades, the development of new anti-cancer drugs has been focused on therapeutic agents that target oncogenic proteins that are specifically mutated or overexpressed in cancer cells. Examples include targeted therapeutics that are directed to block a specific oncogenic pathway (*e.g*. imatinib)^1–3^ or target (*e.g*. trastuzumab)^4,5^ in malignant cells. Included in this focus is the small molecule inhibitor - SI-2 - developed in our laboratory to target steroid receptor coactivator (SRC) members such as SRC-3 (SRC-3/NCOA3/AIB1/RAC3) and SRC-1/2 (SRC-1/NCOA1 and SRC-2/NCOA2/TIF2)^6^ All members of this oncogenic family of coactivators are overexpressed in a broad array of cancer subtypes^7^.

An emerging area for cancer therapy has been focused on the use of immune check point inhibitors to boost immune system for the destruction of tumors^8,9^. Check point blockade is a systemic approach which mostly targets the patient’s immune system, rather than the cancer cells themselves, in order to unleash the immune response and reactivate exhausted T cells to act against cancer cells^10^. Immune check-point blockade has emerged as a game changing approach in clinical oncology for the treatment of selected types of malignancies^9^. Of note are the encouraging survival rates and durability of response in *tough-to-cure* types of cancers such as in advanced metastatic melanomas^11–13^. Meanwhile, in breast and certain other cancers, the response rate for immune checkpoint inhibitors has not been as favorable^14^ in spite of the often significant interaction between breast tumors and the immune system^15–17^. Therefore, a strong impetus exists to design enhancements for the response of breast cancer patients to immunotherapy, possibly by combining it with either standard chemotherapy or targeted cancer drugs. Balancing the direct effects of chemotherapy on the breast tumor and their impact on the anti-cancer activity of the immune system is complex and can possess both beneficial and harmful effects^18,19^.

The beneficiary ‘side effects’ of chemotherapy on the anti-cancer immunity is modeled on an ICD paradigm, which is associated with specific chemotherapeutics and is predicated on the release of certain damage-associated molecular content from dying cancer cells^20,21^. In contrast, harmful effects of chemotherapy on anti-cancer immunity have been associated with the induction of PD-L1 - a central immunoregulatory protein that is expressed in both normal and cancer cells. PD-L1 engages its ligand – programmed death-1 (PD-1) - on activated immune effector cells, and signals the termination of effector cell proliferation and blocks pro-survival cytokine production, resulting in effector T cell death^22–25^. Two predicted recent studies have shown that PD-L1 expression on BC cells is induced following chemotherapeutic treatment^26,27^. In this context, it would be of interest to further assess the impact of different chemotherapeutics on the immunogenicity of BC cells representing different molecular subtypes.

In the present study we exploit a panel of four BC cell lines, representing triple negative breast cancer (TNBC) and ER + types, from both human and mouse species and apply a broad panel of BC small molecule therapeutics to measure the expression of PD-L1 as a result of drug exposure. We demonstrate that the majority of chemotherapeutic agents induce strong expression of PD-L1 as well as other pro-survival genes that are associated with cell stress. We show that a significant decrease in PD-L1 and cell-stress gene expression can be achieved by employing certain combinations of two different agents, which suggests that combinational drug treatment could be beneficial not only for their enhanced potential to directly kill cancer cells, but also as a strategy to effect breast cancer cell killing in a way that evades the immunosuppressive effects of elevated PD-L1 expression and activation of cancer cell pro-survival programs.

## Results

### Chemotherapeutic agents and targeted small molecule agents induce PD-L1 expression in breast cancer cell lines

**Figure 1:**
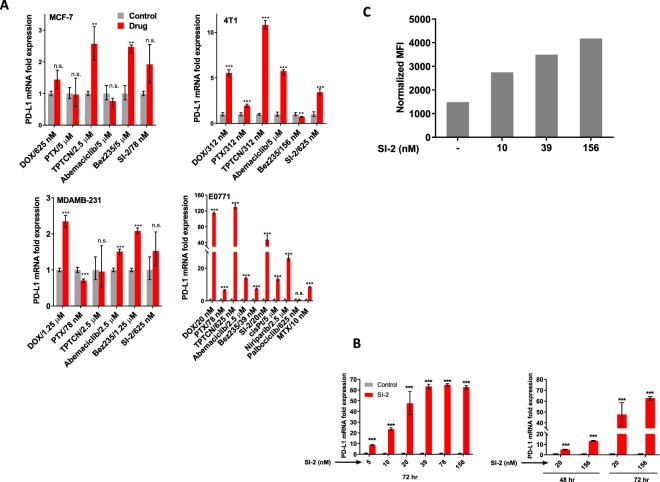
Standard chemotherapeutics, targeted agents and an SRC inhibitor induce PD-L1 expression. (**A**) Cell lines representing either TNBC or ER+ subtypes of BC/mouse mammary carcinoma were treated with cytotoxic concentrations of the mentioned compounds (~IC_50_) and the expression of PD-L1 was determined by qPCR. (**B**) SI-2 induced PD-L1 mRNA expression in E0771 cells in a time and dose dependent manner. Cells were exposed to SI-2 at a concentration of 20 nM and PD-L1 mRNA was quantitated at 48 and 72 hr. (**C**) E0771 cells were treated with SI-2 for 72 hr with the indicated doses of SI-2 and then PD-L1 protein levels on the cell membrane were assessed by flow cytometry. *n.s*., not significant, ***P < 0.005, **P < 0.05, two-tailed Student’s *t*-test.

Recent studies have shown that PD-L1 expression in a variety of cancers is upregulated following exposure to diverse chemotherapeutics with distinct mechanisms of action^26,28–31^. In order to better understand the impact of anti-cancer drug treatment on cancer cell-autonomous expression of PD-L1 in breast/mammary gland cancer, four breast cancer (BC) cell lines - representing both TNBC and ER + – were used; MDA-MB-231 and 4T1 represent TNBC in humans and mice, and MCF-7 and E0771 represent ER + BC in humans and mice. The cells have been treated with a panel of six drugs/drug candidates with distinct mechanisms of inhibitory activity: doxorubicin (DOX), paclitaxel (PTX), Abemaciclib (ABE), Topotecan (TPTCN), BEZ235 and SI-2 representing respectively a topoisomerase-2 inhibitor, microtubulin inhibitor, CDK (cyclin dependent kinase)4/6 inhibitor, topoisomerase-1 inhibitor, PI3K-mTOR dual inhibitor and SRC-3 inhibitor^6^. Following exposure to a cytotoxic dose of each molecule – which was set at ~50% growth inhibition (GI) concentration (Fig. S1) – PD-L1 mRNA induction was observed in an overwhelming majority of cases (Fig. 1A). Because it can be used as an aggressive and ER + immunocompetent *in vivo* tumor model – E0771 cells were tested with additional molecules: cis-platin (cisPt), Palbociclib, Niraparib and methotrexate (MTX). Among the cell lines that we tested, E0771 is the most responsive model in terms of induced PD-L1 expression as a result of drug treatment. Among the tested molecules, DOX and TPTCN brought about the highest amounts of PD-L1 expression in E0771 cells. SI-2 was found to be the next strongest inducer of PD-L1 mRNA expression. Treatment of E0771 cells with SI-2 resulted in a time- and dose-dependent PD-L1 mRNA induction (Fig. 1B), which correlates with cell surface expression of PD-L1 (Fig. 1C). Collectively this data shows that PD-L1 is induced in E0771 breast cancer cells proportionally to the intensity of drug pressure.

### Drug induced PD-L1 expression takes place through a cellular stress response pathway

**Figure 2:**
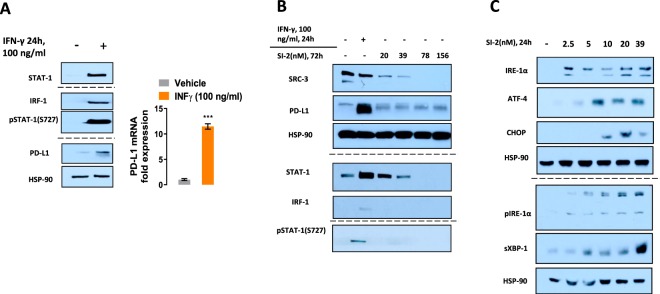
Stimulation of PD-L1 expression with IFN-γ results in activation of IFN-γ related signaling pathway components. (**A,B**) E0771 cells were treated with 100 ng/ml IFN-γ and Western analyses for IRF-1, STAT1, pSTAT1 (S727), PD-L1 and HSP-90 was performed indicated that IFN-γ stimulated canonical components of the IFN-γ signaling pathway. In contrast, treatment of E0771 cells with SI-2 induced PD-L1 without elevating pSTAT1 or IRF-1. (**C**) SI-2 induced the expression of cell stress response proteins ATF4, IRE1α, p-IRE1α and XBP1s. E0771 cells were treated for 24 hr with the indicated concentrations of SI-2. ***P < 0.005, two-tailed Student’s *t*-test. For each group of WB figures (**A–C**): all the blots were performed in the same experimental setup and processed in parallel. All gels were loaded with identical amount of protein sample. Dashed separating lines indicate that the blots were run in parallel but on separate gels. Full length blots are available in the supplementary information.

It has been reported that PD-L1 functions not only as a key regulator of T-cell exhaustion, but that it also has a cancer cell autonomous pro-survival function that can promote tumor metastasis and drug resistance^32–35^. To evaluate whether the overexpression of PD-L1 is taking place within the broader context of a cell stress survival program, we compared the quasi-physiological mode of PD-L1 induction with interferon-γ (IFN-γ) with drug-related induction. Under physiological conditions, IFN-γ signaling is reported to act through the JAK-STAT1-IRF1 axis to exert robust PD-L1 induction, whereas loss-of-function mutilations in JAK1/2 are correlated with loss of PD-L1 expression^36,37^. In our experimental model, IFN-γ-induced PD-L1 expression also was found to take place through the conventional JAK-STAT1-IRF1 pathway (Fig. 2A,B). However, during the induction of PD-L1 with anti-cancer compounds, upstream markers of the JAK-STAT1-IRF pathway were absent (Fig. 2B), suggesting that under stressful conditions there is an alternative pathway for PD-L1 expression, similar to an alternative cytokine signaling independent inflammation^38^.This form of cell stress-related, but not IFN-γ signaling context PD-L1 expression, could be associated with the emerging role of PD-L1 as a cell autonomous factor that is responsible for cancer cell survival and resistance to drug treatment^32,39,40^. This assumption is supported by the observed drug-induced upregulation of cell stress markers (Fig. 2C) which are known to be involved in oncogenic, cancer cell pro-survival processes^35,41,42^.

### PD-L1 induction and cell stress are concomitantly regulated by drug combination

**Figure 3:**
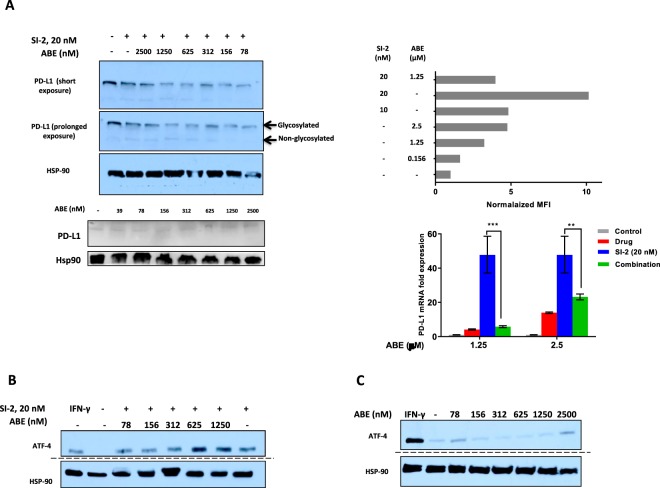
Combined treatment with the CDK4/6 inhibitor abemaciclib (ABE) and SI-2 can be strongly cytotoxic without inducing PD-L1 expression. (**A**) E0771 cells were treated with SI-2 or ABE or both for 72 hr at the indicated concentrations. Increased concentrations of ABE result in reduced PD-L1 expression compared to SI-2 alone, on protein (top) and mRNA (bottom right) levels. Compared to SI-2, cytotoxic concentrations of ABE alone do not bring about strong induction of PD-L1 mRNA (bottom right) or protein levels (top right, bottom left). (**B**) Addition of up to 625 nM ABE to SI-2 does not induce ATF4 expression compared with SI-2 alone. Western analysis of ATF-4 protein expression was determined 24 hr after treatment with the indicated concentrations of SI-2 and/or ABE. (**C**) Exposure of E0771 cells to cytotoxic concentrations of ABE for 24 hr only moderately induces the expression of ATF4. ***P < 0.005, **P < 0.05, two-tailed Student’s *t*-test. For each group of WB figures (**A–C**): all the blots were performed in the same experimental setup and processed in parallel. All gels were loaded with identical amount of protein sample. Dashed separating lines indicate that the blots were run in parallel but on separate gels. Full length blots are available in the supplementary information.

Our results shown above demonstrate that each small-molecule drug has a different impact on the magnitude of PD-L1 mRNA induction (Fig. 1A) with fold change values at the mRNA level in E0771 cells varying from ~10 fold (PTX and ABE) to ~10^2^ fold (DOX and TPTCN). Intrigued by these differences, we tested the effects of a combination treatment with strong and weak PD-L1 inducers to explore which one of them possesses the dominant impact on PD-L1 induction. To this purpose, SI-2 (strong) and ABE (weak) were combined for the first test. Surprisingly, combined treatment with these two drugs did not produce an additive effect on PD-L1 induction but rather resulted in PD-L1 expression levels that resembled the weaker PD-L1 inducer - ABE (Fig. 3A top and 3A bottom right), without reducing cancer cell cytotoxicity (Fig. S2). Exposure of cells to ABE alone at a range of concentrations did not change PD-L1 levels -which remained similar to the untreated control cells (Fig. 3A bottom left). These observations indicate that by itself, ABE has a neutral to moderate-inducing effect on protein expression of PD-L1 but is able to suppress PD-L1 overexpression that would be otherwise induced by SI-2. Bearing in mind the possibility that under pharmacological pressure, PD-L1 expression might be associated with activation of stress-related survival programs, we tracked the expression of the stress marker activating transcription factor 4 (ATF4) under conditions of combinatorial ABE/SI-2 treatment and found that the addition of ABE - up to 625 nM - to SI-2 doesn’t result in further upregulation of ATF4 (Fig. 3B). Moreover, exposure of E0771 cells to lethal doses of ABE alone (up to 1.25 µM), didn’t upregulate the expression of ATF4 (Fig. 3C).

These observations indicate that cell stress pathway induction could be uncoupled from cancer cell cytotoxicity by choosing specific drug combinations. This suggests that it might be possible to better kill malignant cells through an appropriate combination of drugs and administration regimen that does not activate PD-L1 expression or cellular stress pathways. We refer to this fashion of cell death of the cancer cell a ‘silent death’ which might be desirable for killing cancer cells without enacting the immunosuppressive effects of PD-L1 expression. Further investigation of the mechanisms by which some anti-cancer agents induce cytotoxicity without inducing PD-L1 expression or cellular stress pathways is needed to understand this process fully at a molecular level.

### PD-L1 induction is transient and not associated with cancer cell acquired resistance to drug pressure

**Figure 4:**
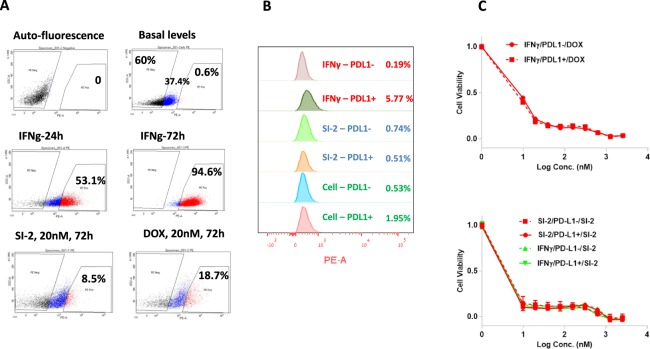
Cell populations expressing elevated PD-L1 levels do not acquire short-term resistance to SI-2. (**A**) E0771 cells were treated with either IFN-γ (24 hr) or SI-2 or DOX (72 hr) at the indicated concentrations that then sorted into PD-L1 negative and PD-L1 positive populations. (**B**) After allowing cells to recover from compound exposure for seven days, PD-L1 levels were assessed again by flow cytometry, revealing that PD-L1 expression returned to basal levels of expression. (**C**) Cells from both the PD-L1 negative and positive populations treated with either SI-2 or IFN-γ were treated again with a dose range of SI-2 or doxorubicin (DOX) after the seven day recovery period and their viability was determined by MTS assay.

Recent studies have demonstrated that there is a correlation between PD-L1 expression and cancer cell intrinsic drug resistance^43^. In order to investigate this correlation in greater detail in our BC cell line models, we induced PD-L1 overexpression in E0771 cells either pharmacologically (by SI-2 or DOX) or by mimicking physiological conditions (exposure to IFN-γ). Following treatment, the cells were sorted into PD-L1^hi^ and PD-L1^neg^ populations (Fig. 4A) and cultured for a recovery period of seven days after which both groups were measured for PD-L1 expression levels again. After the recovery period, PD-L1 levels in cells which initially had high PD-L1 expression return to the basal expression state (Fig. 4B). To test the possibility that transient PD-L1 induction is a marker for the activation of a stable, long-term pro-survival program, we performed viability assays, which indicated that PD-L1^hi^ and PD-L1^low^ cells respond similarly to drug treatment, either SI-2 or DOX, independently on their initial PD-L1 expression levels (Fig. 4C). These observations indicate that PD-L1 induction is more likely to take place and to remain highly elevated under ‘continuous’ stressful conditions.

## Discussion

Expression of the immunosuppressive PD-L1 protein on the surface of cancer cells has been established as having a major role in the ability of malignant cells to evade immune responses^23,44,45^. Moreover, several studies have shown that PD-L1 upregulation in cancerous and pre-malignant cells possesses non-immune functionalities associated with cancer-initiation, tumor cell-intrinsic cell survival and tumor progression^32,46–48^. In this context, the upregulation of PD-L1 by cancer cells in response to drug exposure implies that this is part of a pro-survival program. In agreement with previous studies^26,27^, here we show that PD-L1 is overexpressed in cancer cells as a result of treatment with a variety of anti-cancer agents; exposure of BC cell lines to various small-molecule therapeutics resulted in a general phenomenon of PD-L1 upregulation, through a non-canonical signaling pathway that does not involve IFN-γ. However, from our cell-based experiments, we found that drug-induced PD-L1 overexpression didn’t result in the acquisition of intrinsic cancer cell resistance to a second round of drug treatment. Indeed, after the removal of drug pressure, PD-L1 expressed returned to a low, basal level. This observation suggests that it might be possible to time the delivery of chemotherapeutic treatment with immune checkpoint inhibitors or other anti-cancer agents in a way that results in a more optimal outcome. We also show that it might be possible, through the pre-selection of appropriate cancer drug combinations, to achieve cancer cell growth inhibition in a way that does not trigger highly elevated PD-L1 expression. We speculate that many/most current standard-of-care therapies for BC are likely to elevate PD-L1 expression. Because the transient induction of this immunosuppressive protein only occurs in response to drug stress-pressure, pre-intervention analyses of BC biopsies fail to adequately account for the dynamic expression of PD-L1 during chemotherapy. Testing of chemotherapeutics, targeted therapeutics and checkpoint inhibitors in neoadjuvant window-of-opportunity studies may be useful to find ways to promote BC tumor cell cytotoxicity without inducing PD-L1 or other pro-survival stress related pathways.

## Conclusions

Strong induction of PD-L1 expression was observed in a panel of BC cells exposed to various small molecule anti-cancer drugs. Drug-induced PD-L1 expression was taking place in the context of cell stress, implying that its activation is part of a pro-survival program in response to drug pressure. We found however, that the activation of the cell-intrinsic pro-survival programs, which manifested through cell-autonomous induction of PD-L1 and upregulation of cell stress pathways, could be avoided with specific drug-combinations. We demonstrated the concept of such as drug-combination by utilizing the SRC-3 inhibitor SI-2 alongside the CDK4/6 inhibitor abemaciclib. This combination resulted in a cancer-cell killing effect that was accompanied by only moderate upregulation of PD-L1 and cell-stress markers. The ability of ABE to suppress PD-L1 expression levels might be attributed to its ability to induce cell cycle arrest at G1^49^. We refer to this effect as a ‘silent death’ to point out that it is possible to achieve potent BC cytotoxicity while also preventing BC tumor cells from activating immunosuppressive and pro-survival programs which could latter lead to the expansion of drug-resistant populations of cancer cells. We also found that the induction of PD-L1 that arose from the anti-cancer agents that we tested occurred outside of the established IFN-γ pathway and is not specific to inhibition of the steroid receptor coactivator signaling.

## Materials and Methods

### Cell culture

E0771, 4T1, MCF-7 and MDAMB-231 cell lines were obtained from American type culture collection (ATCC). E0771, MCF-7 and MDA-MB-231 cells were maintained in Dulbecco’s modified eagle media (DMEM). 4T1 cells were maintained in Roswell park memorial institute (RPMI) medium. All media were supplemented with 10% fetal bovine serum (FBS), 1% Glutamax (ThermoFisher 35050061) and 1% penicillin/streptomycin. Cells were grown at 37 °C under 5% CO_2_ atmosphere. Stimulation with Interferon gamma (IFN-γ) was performed by treating cells for 24 hr with 100 ng/ml recombinant human (Biolegend #570206) or mouse (Biolegend #575306) IFN-γ for human or mouse cell lines, respectively.

### Cell viability assay

Cells were seeded on 96 well plates (E0771 and MDAMB-231 at 3,000 cells/well, 4T1 at 6,000 cells/well and MCF-7 at 10,000 cells/well) and allowed to adhere overnight. Media was then removed and cells were provided with fresh compound(s)-containing media. After indicated periods of treatment, drug-containing media was replaced with fresh media containing MTS ([3-(4,5-dimethylthiazol-2-yl)-5-(3-carboxymethoxyphenyl)-2-(4-sulfophenyl)-2H-tetrazolium, inner salt]) reagent (Promega cat no. G3582) and then the cells were incubated for an additional 1–4 hr. MTS absorbance measurements were then performed with a Multiskan FC Microplate Photometer plate reader (ThermoFisher) at 490 nm. After blank (media only) normalization, cell viability was calculated relative to vehicle-treated cells. Each point reflects at least four replicates.

All commercial small molecule drugs were obtained from MCE. SI-2 was synthesized as previously described^6^.

### Immunoblotting

Whole cell lysates were obtained using RIPA Lysis and extraction buffer (ThermoFisher, cat no. 89900). Following ice-cold incubation with extraction buffer for 30 min, the lysates were centrifuged at 4 °C, 15000 rpm for 15 min after which the supernatants were collected and protein concentrations were calculated using the Pierce BCA (bicinchoninic acid assay) protein assay kit (ThermoFisher, cat no. 23225). The supernatants were proportionally mixed with 4x Laemmli sample buffer (BioRad, cat no. 1610747) containing 10% 2-mercaptoethanol and boiled for five min at 95 °C. Equal protein amounts were brought to equal volumes with sample buffer and run on 4–20% precast polyacrylamide gels (BioRad Mini-PROTEAN TGX Precast Protein Gels). The protein samples were transferred to nitrocellulose membranes using an iBlot Gel Transfer Device (ThermoFisher), blocked with PBS (phosphate-buffered saline)-Tween buffer, containing 5% nonfat milk dissolved powder for 60 min. After this, relevant primary antibodies were added and the membranes were incubated over-night at 4 °C (Table S1). After thorough washing with PBS, the membranes were incubated at room temperature for 60 min in PBS-Tween buffer containing 5% nonfat milk supplied with a relevant HRP (horseradish peroxidase)-conjugated secondary antibody. Membranes were then washed three times with PBS and protein bands were detected with either enhanced chemiluminescence (ECL) substrate (ThermoFisher, cat no. 32106) or Pico PLUS Chemiluminescent substrate (ThermoFisher, cat no. 34580).

### Flow cytometry

Cells were harvested, washed with PBS and the cell pellet was obtained by brief centrifugation. Cell pellets was then rinsed in 100–200 μL staining buffer (BioLegend, cat no. 420201) and PE-conjugated CD274 antibody was added (BioLegend, cat no. 124308, 1/100). Following 45–60 min incubation on ice, cells were washed with PBS and resuspended in 0.5–1 ml staining buffer. Before subjecting cells to FACS analysis, all the samples were passed through a 0.40 μm filter and additionally stained with DAPI (4′,6-diamidino-2-phenylindole, Invitrogen, cat no. R37606), except for auto-fluorescence control samples. For all samples a ‘full minus one’ (FMO) control was performed^50^. Samples were run on a LSRII cell analyzer (Becton and Dickinson) and sorted with a FACSArial cell sorter (Becton and Dickinson) using the Diva software package (Becton and Dickinson). Analyses were performed with either Diva or FlowJo_v10 software. Normalized MFI (mean fluorescent intensity) was calculated for all samples relatively to the untreated control group. Graphs were generated with Prism 7 software (GraphPad Software, Inc.).

### Real time quantitative polymerase chain reaction (RT qPCR)

RNA was extracted from cells using TRIzol reagent (Invitrogen). Reverse transcription cDNA synthesis was performed with the VILO SuperScript cDNA Synthesis Kit (Invitrogen) using 2 μg of total RNA. Probe-based real time quantitative PCR was performed using Universal Probe Library probes (Roche) and TaqMan Universal Master Mix (Applied Biosystems) on a StepOnePlus Real-Time PCR machine (Applied Biosystems). Primers were designed using the *Roche* ‘universal probe library assay design center system’ (Table S2). Relative mRNA expression of *CD274* was calculated by the ΔΔCT method with normalization to *GAPDH* or *ACTB*. Each result is represented by SD of at least three technical replicates.

## Supplementary information

Supplementary info.
