# Author Correction: Drug-induced PD-L1 expression and cell stress response in breast cancer cells can be balanced by drug combination

**DOI:** 10.1038/s41598-020-60964-w

**Published:** 2020-03-05

**Authors:** Yosi Gilad, Yossi Eliaz, Yang Yu, Sang Jun Han, Bert W. O’Malley, David M. Lonard

**Affiliations:** 1 Department of Molecular and Cellular Biology, Baylor College of Medicine, Houston, TX USA; 2 Department of Molecular and Human Genetics, Baylor College of Medicine, Houston, TX USA

Correction to: *Scientific Reports* 10.1038/s41598-019-51537-7, published online 22 October 2019

In Figure 2A, IFN is incorrectly spelt as INF. The correct Figure 2 appears below as Figure 1.

**Figure 1:**
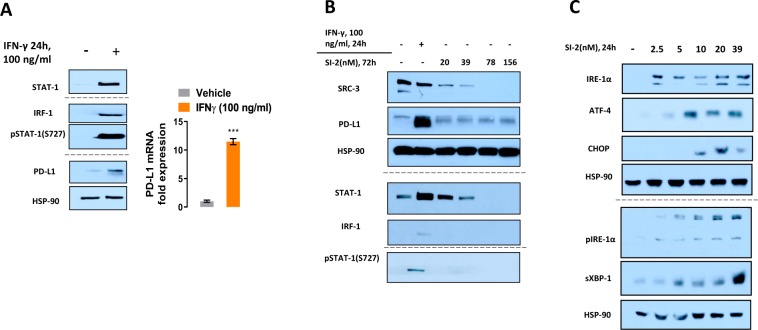
.

