# Supplementary material for: Drug-induced PD-L1 expression and cell stress response in breast cancer cells can be balanced by drug combination
Source: Sci Rep. 2019 Oct 22;9:15099. doi: 10.1038/s41598-019-51537-7 (PMC6805932; doi:10.1038/s41598-019-51537-7)
Supplement: Supplementary file 1 — Supplementary info. [file 41598_2019_51537_MOESM1_ESM.pdf]

Drug-induced PD-L1 expression and cell stress response in breast cancer cells can be balanced by drug combination

Yosi Gilad[1], Yossi Eliaz[2], Yang Yu[1], San Jung Han[1], Bert W. O'Malley[1]* and David M. Lonard[1]*

[1]Department of Molecular and Cellular Biology, Baylor College of Medicine, Houston, Tx, USA [2]Department of Molecular and Human Genetics, Baylor College of Medicine, Houston, Tx, USA

Contact information: *Corresponding authors. Mailing address: Department of Molecular and Cellular Biology, Baylor College of Medicine, One Baylor Plaza, Houston, TX 77030. Tel.:(713) 798-6205; fax: (713) 798-5599. E-mail address: berto@bcm.edu (BWO); Department of Molecular and Cellular Biology, Baylor College of Medicine, One Baylor Plaza, Houston, TX 77030. Tel.:(713) 798-6258; fax: (713) 790-1275. E-mail address: dlonard@bcm.edu (DML)

Running title: SRC inhibitors and anti-cancer agents induce PD-L1 expression in cancer cells

Table S1 – Antibodies

Antibody	Source	Catalogue no.	Clone	Dilution
PD-L1	CST	13684	E1L3N	1/500
SRC-3	CST	2126	5-E11	1/1000
ATF-4	CST	11815	D4B8	1/1000
IRF-1	CST	8478	D5E4	1/500
XPB-1s	CST	12782	D2C1F	1/500
PERK	CST	3192	C33E10	1/1000
STAT1	CST	14994	D1K9Y	1/500
pSTAT1(S727)	BioLegend		A15158B	1/500
IRE-a	CST	3294	14C10	1/500
pIRE-a(S724)	Invitrogem	PA1-16927	Polyclonal	1/500
HSP90	CST	4877	C45G5	1/1000
HRP-Conjugated anti rabbit IgG	CST	7074		1/5000
HRP-Conjugated anti mouse IgG	CST	7076		1/5000

Table S2 – primers and probes (from Roche universal probe library) used for qPCR

Mouse primers

Gene	Fwd.	Rev.	Probe
GAPDH	GGG TTC CTA TAA ATA CGG ACT GC	CCA TTT TGT CTA CGG GAC GA	52
ACTB	CCA ACC GTG AAA AGA TGA CC	ACC AGA GGC ATA CAG GGA CA	64
CD274	TCC ATC CTG TTG TTC CTC ATT	TCC ACA TCT AGC ATT CTC ACT TG	101

Human primers

Gene	Fwd.	Rev.	Probe
GAPDH	TCC ACC CAT GGC AAA TTC	TCC ACC CAT GGC AAA TTC	9
ACTB	TCC CCC AAC TTG AGA TGT ATG	ACT GGT CTC AAG TCA GTG TAC AGG	71
CD274	CAG TTC TGC GCA GCT TCC	TTC AGC AAA TGC CAG TAG GTC	28

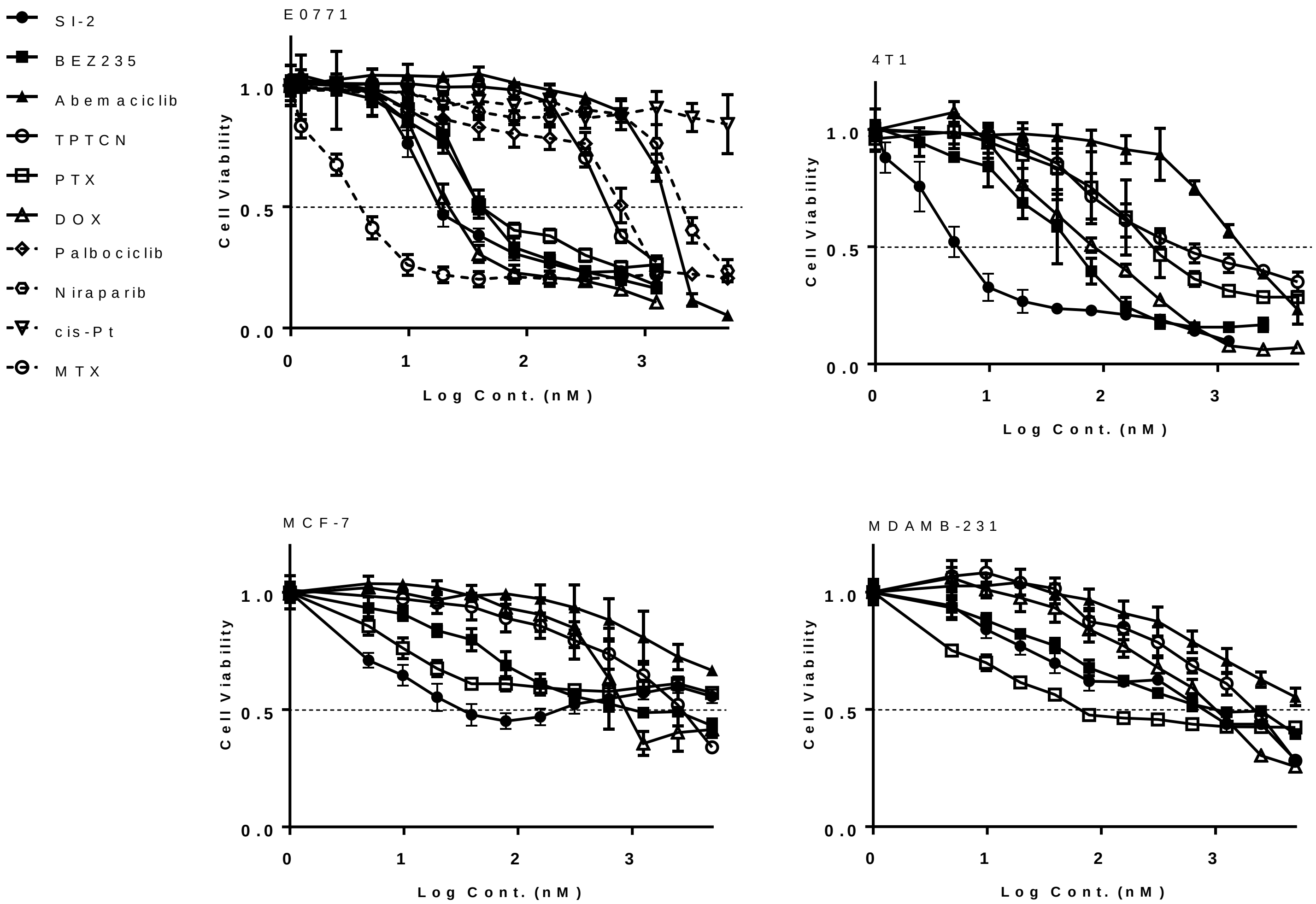

Fig. S1

Cytotoxicity assays: Cells were treated with compounds for 72 hr followed by assessment of cell viability using an MTS assays.

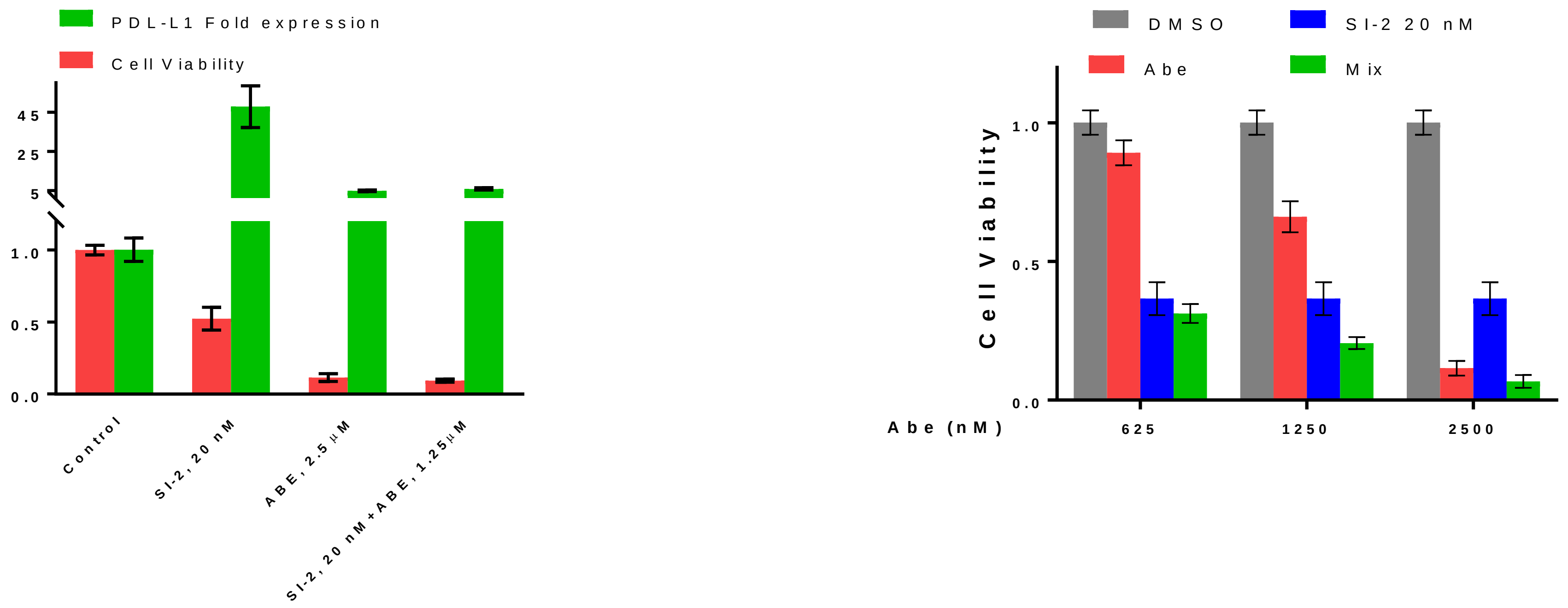

Fig. S2

Combined SI-2 and ABE treatment results in downregulation of PD-L1 without impairing cancer cell cytotoxicity. E0771 cells were treated with SI-2 or ABE alone or in combination for 72 hr followed by assessment of cell viability by MTS assays and PD-L1 mRNA expression levels by qPCR.

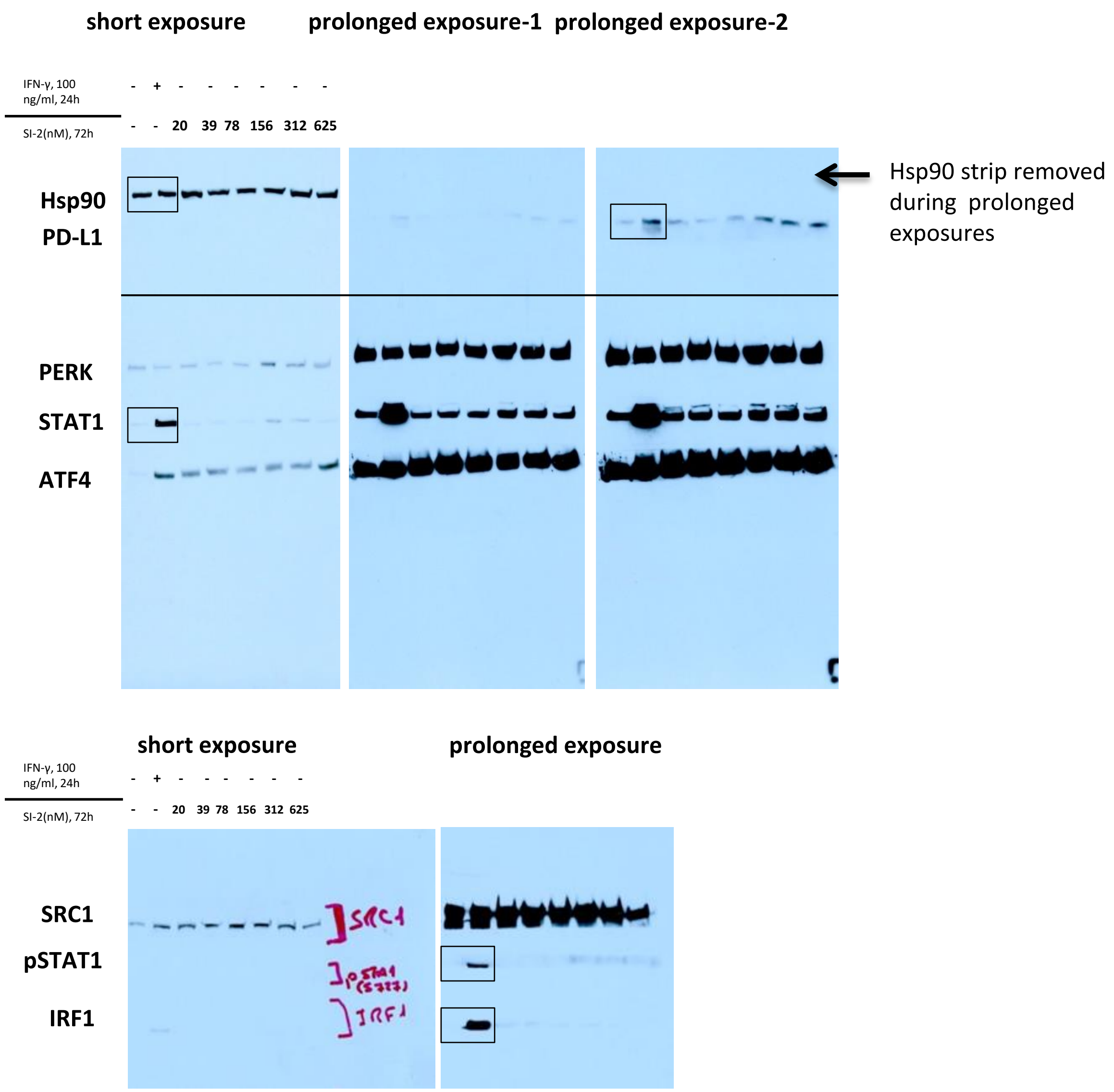

Fig. 2a – full length blots. Portions in black frames indicate the samples presented in the main article. All blots in this figure were performed in the same experimental setup and processed in parallel. All gels were loaded with identical amount of protein sample. Black separating line indicates that the blots run in the same tank on separate gels.

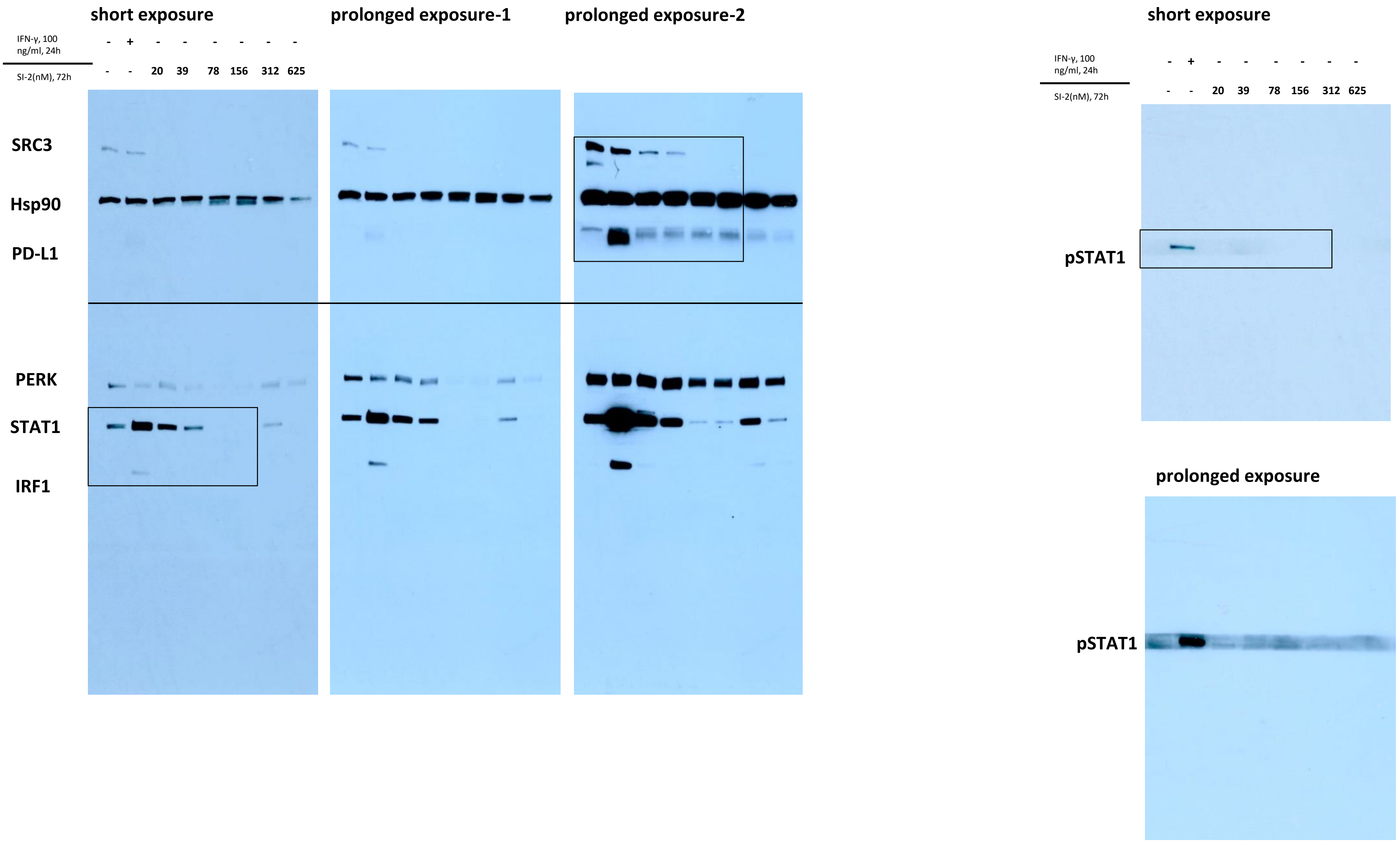

Fig. 2b – full length blots. Portions in black frames indicate the samples presented in the main article. All blots in this figure were performed in the same experimental setup and processed in parallel. All gels were loaded with identical amount of protein sample. Black separating line indicates that the blots run in the same tank on separate gels.

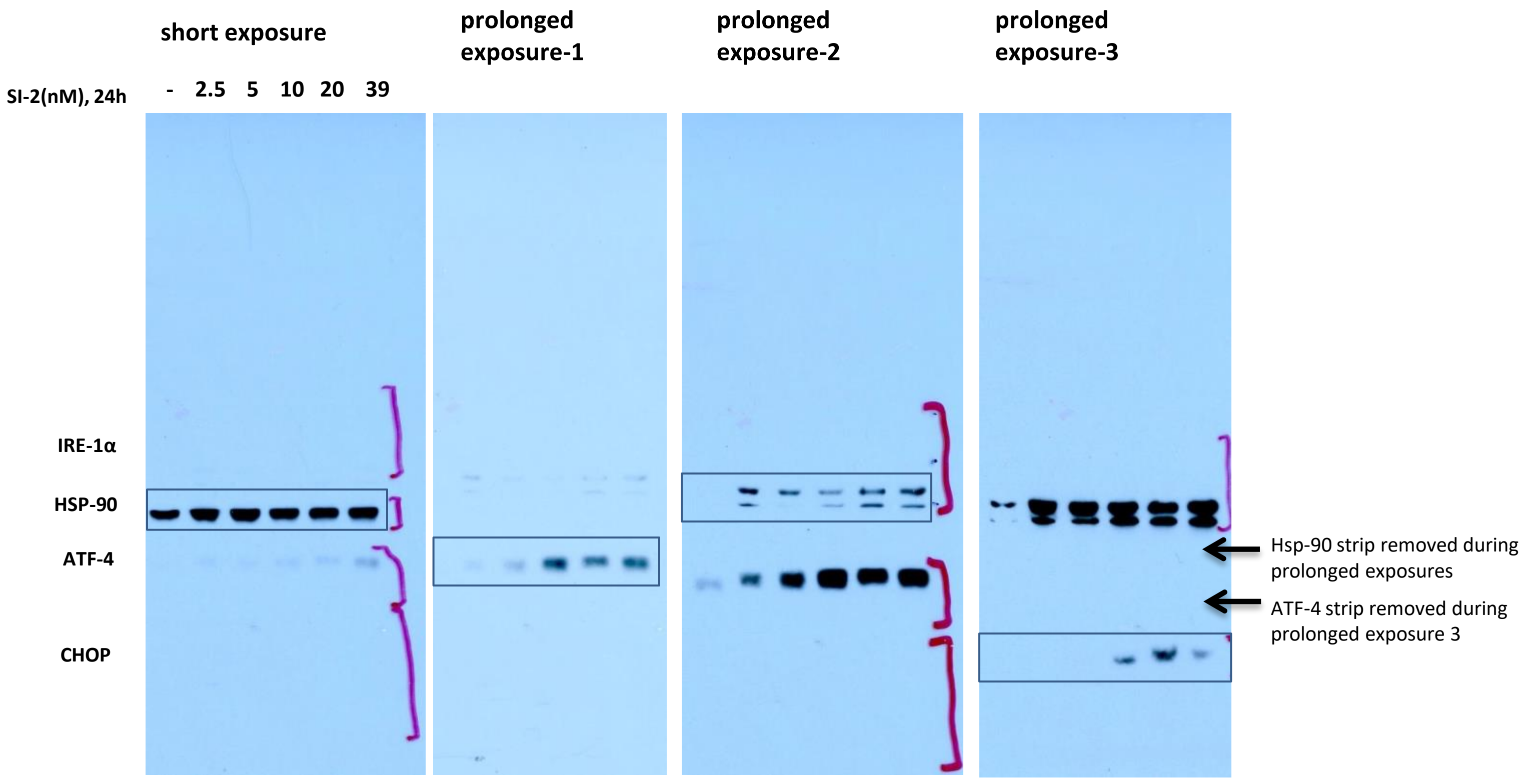

Fig. 2c – full length blots. See legends on the next slide

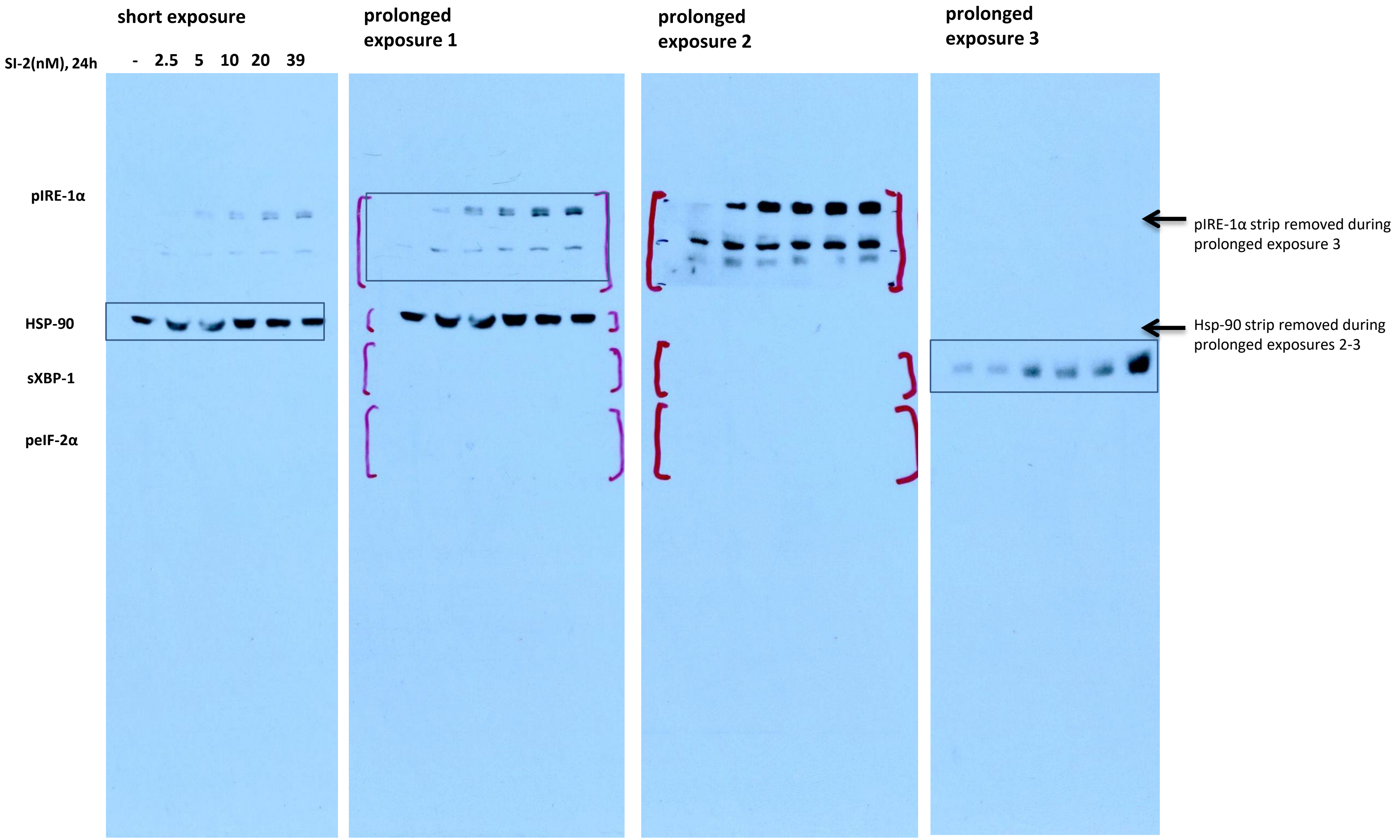

Fig. 2c– full length blots (cont.). Portions in black frames indicate the samples presented in the main article. All blots in this figure were performed in the same experimental setup and processed in parallel. All gels were loaded with identical amount of protein sample. Black separating line indicates that the blots run in the same tank on separate gels.

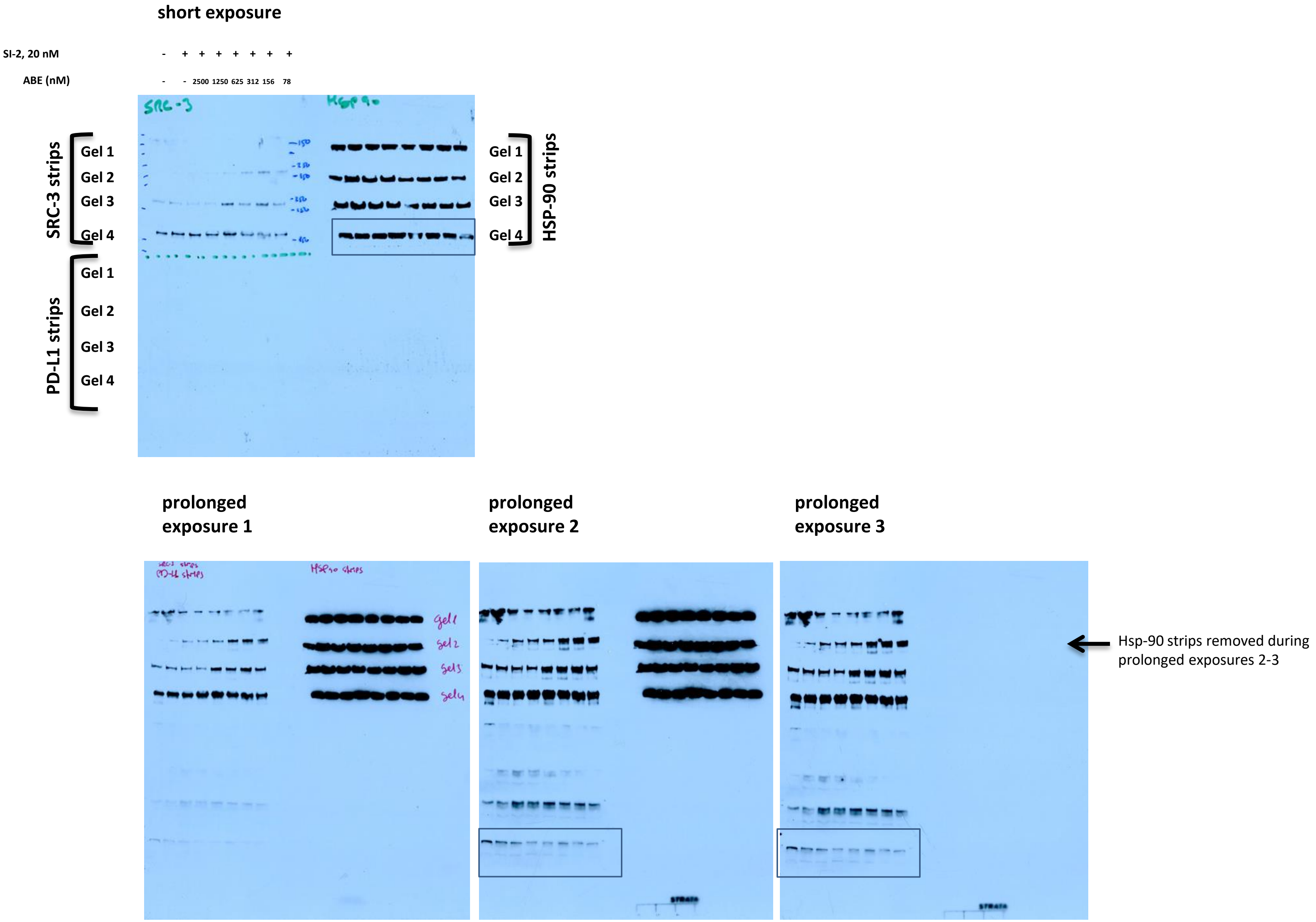

Fig. 3a top - full length blots. Portions in black frames indicate the samples presented in the main article. All blots in this figure were performed in the same experimental setup and processed in parallel. All gels were loaded with identical amount of protein sample. Black separating line indicates that the blots run in the same tank on separate gels.

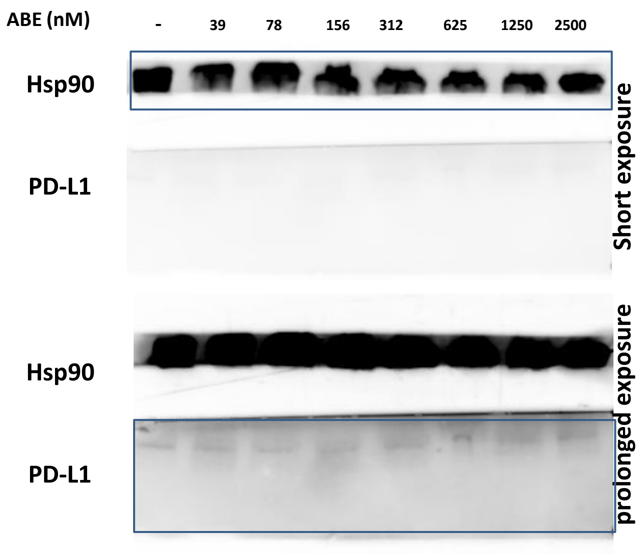

Fig. 3a bottom – full length blots. Portions in black frames indicate the samples presented in the main article. All blots in this figure were performed in the same experimental setup and processed in parallel. All gels were loaded with identical amount of protein sample.

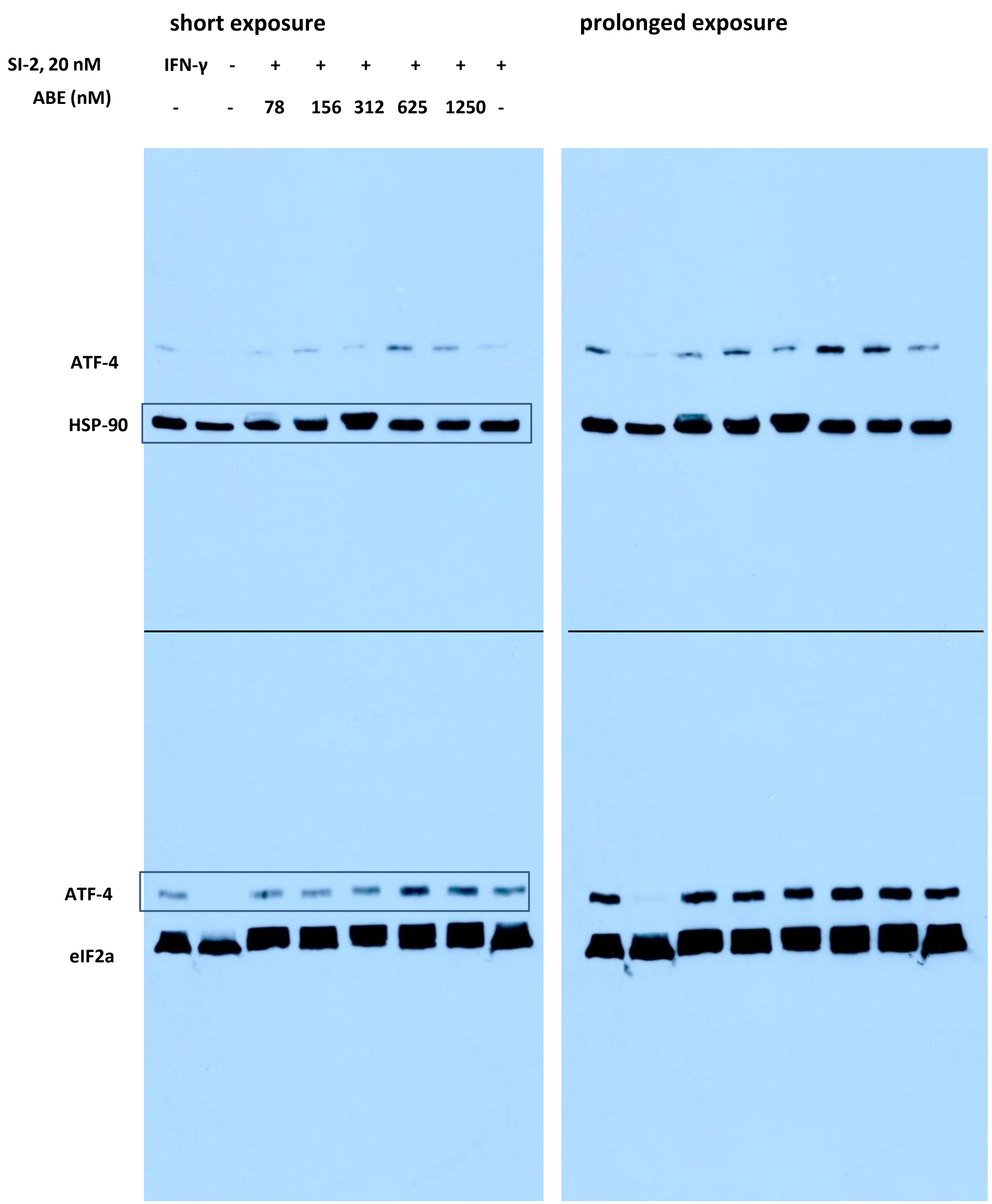

Fig. 3b – full length blots. Portions in black frames indicate the samples presented in the main article. All blots in this figure were performed in the same experimental setup and processed in parallel. All gels were loaded with identical amount of protein sample. Black separating line indicates that the blots run in the same tank on separate gels.

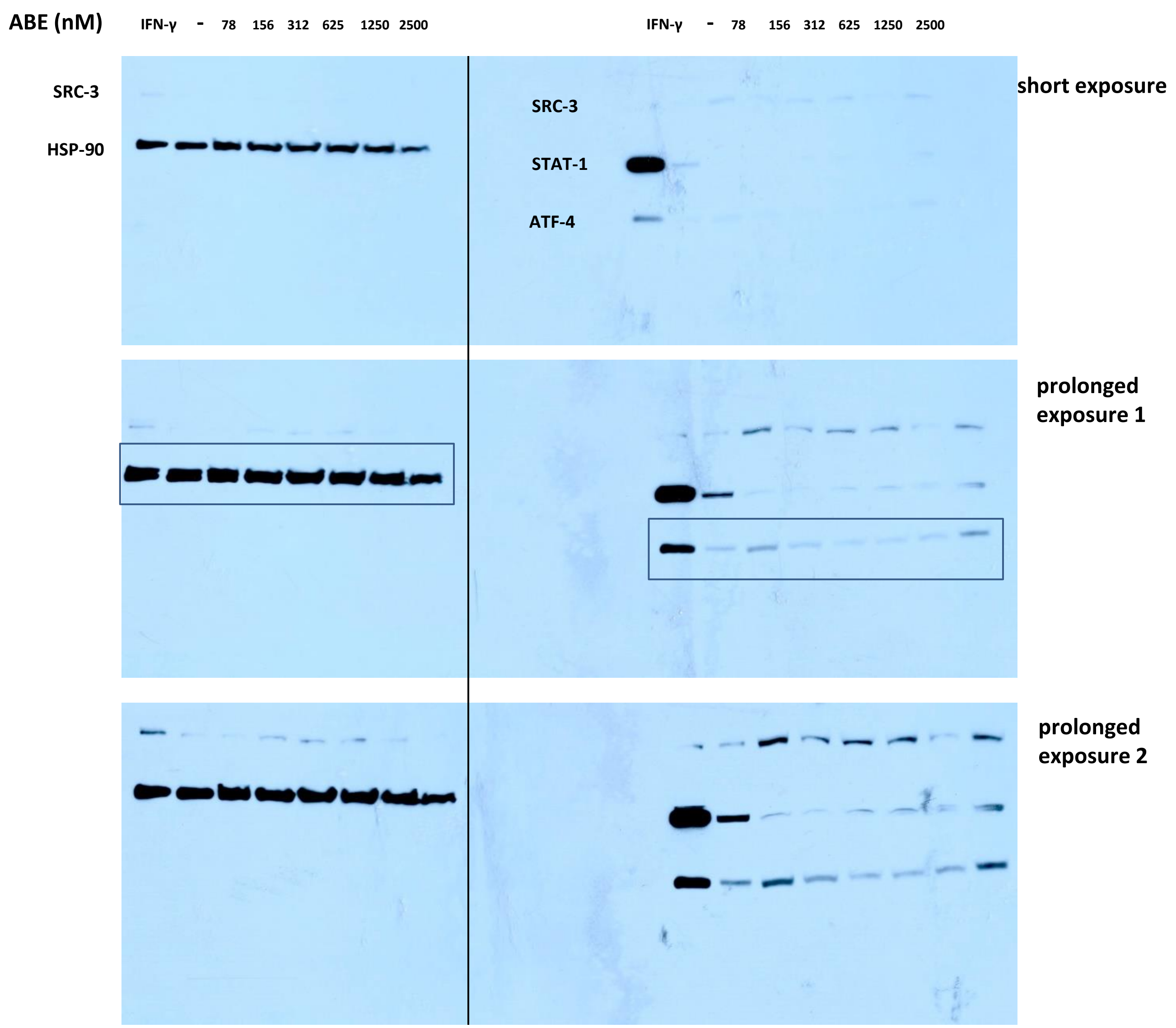

Fig. 3c – full length blots. Portions in black frames indicate the samples presented in the main article. All blots in this figure were performed in the same experimental setup and processed in parallel. All gels were loaded with identical amount of protein sample. Black separating line indicates that the blots run in the same tank on separate gels.
